# Four years of development as a gathering place for international researchers and readers in STEM education

**DOI:** 10.1186/s40594-018-0153-0

**Published:** 2018-12-21

**Authors:** Yeping Li

**Affiliations:** Texas A&M University, College Station, TX 77843-4232 USA

## Introduction

The publication of five articles at the end of August 2014 marked the official launch of the *International Journal of STEM Education*. At the end of July 2018, the journal had completed four publication cycle years, that is, publication cycle 1 (PC-1): August 2014 – July 2015, publication cycle 2 (PC-2): August 2015 – July 2016, publication cycle 3 (PC-3): August 2016 – July 2017, and publication cycle 4 (PC-4): August 2017 – July 2018. Along with the rapid development of STEM education internationally, the journal has also established itself in the global field. Now is therefore a fitting time to briefly review and reflect on (1) the journal’s overall performance over the past four publication cycle years, (2) the journal’s publications during these four publication cycle years, and (3) the journal’s position in comparison with other journals in STEM education.

## How well has the journal performed over the four publication cycle years?

One critical aspect of an academic journal’s performance is to look at the impact of the journal as a whole. Although there are different measures that can be used in a professional field to assess a journal’s impact (e.g., peer evaluation, recognition within the field, and inclusion by various indexing services), one commonly valued indicator is the number and type of indexing services that include the journal. After a journal has obtained an Impact Factor, scholars often want to know its value.

Beginning in early 2018, the *International Journal of STEM Education* has been selected and reviewed in the Web of Science’s Emerging Sources Citation Index (ESCI), in addition to other important professional services including SCOPUS, Google Scholar, and almost 20 other data searching and indexing services. Being accepted into ESCI is a first step in the evaluation process for obtaining an Impact Factor, which is exclusively published for journals included in Science Citation Index Expanded (SCIE) or Social Sciences Citation Index (SSCI) (Testa, 2018). It is certainly a very important step. Inclusion in ESCI means that the journal has been identified as important to key opinion leaders, funders, and evaluators worldwide. ESCI allows researchers to discover new areas of research in evolving disciplines, as well as relevant interdisciplinary scholarly content across rapidly changing research fields. The journal records are now covered in the Web of Science, which means that articles are discoverable there with full citation counts, author information and other enrichments. Given the journal’s short publication history, this coverage is indeed a marvelous achievement! This achievement reflects high quality of the articles that authors have contributed in the internationally oriented field of STEM education during the past four publication cycle years.

## What has the journal published during its first four publication cycle years (August 2014 through July 2018)?

A journal’s performance is related closely to its ability to attract and publish high-quality papers. It can be measured in part by the number of papers published in each cycle year and how well these publications are received and cited by scholars in the field. In this editorial, I will focus on the number of items published in the journal’s first four cycle years.

**Fig. 1:**
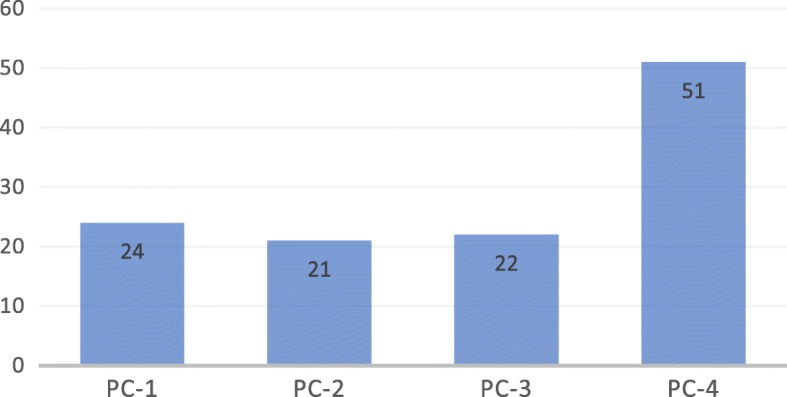
Number of publications by publication cycle year

The *International Journal of STEM Education* has published a total of 118 items over the past four publication cycle years, thus averaging 29 items per publication year. Figure 1 shows the journal’s growth in terms of the number of items published for each publication cycle year, from August 2014 to July 2018. It is clear that the journal started to receive and publish many more articles since August 2017 (51 publications in the fourth cycle), in comparison to its prior three publication cycles from August 2014 to July 2017 (i.e., 24, 21, and 22 in the first, second, and third cycles, respectively).

Because the journal publishes several types of articles, Table 1 shows the breakdown of these 118 publications by type for each publication cycle year.

**Table 1:** Number (percentage) of publications of each type by publication cycle year

	PC-1	PC-2	PC-3	PC-4	TOTAL
Research articles	18 (75%)	15 (71%)	19 (86%)	39 (76%)	91 (77%)
Research reviews	2^b^ (8%)				2 (2%)
Short reports	1 (4%)	3 (14%)	2 (9%)	6 (12%)	12 (10%)
Commentaries	2 (8%)	2 (10%)	1 (5%)	3 (6%)	8 (7%)
Others ^a^	1 (4%)	1 (5%)		3 (6%)	5 (4%)

^a^include editorials, guest editorials, and errata

^b^two articles were submitted and published as “research”, but they are “reviews”

Regarding types of publications, seen in Table 1 the journal has shown a consistent pattern over the four publication years. The journal consists mainly of research articles, followed by short reports and commentaries. There is a noticeable shortage of research reviews. Although the relatively short history of STEM education may be one reason for such a shortage, the journal certainly wants to encourage submission of many more research reviews.

In addition to the number of publications, we next consider the subject discipline(s) on which the articles have focused. Specifically, our journal aims to serve as a multidisciplinary education journal that spans disciplinary boundaries. Thus, we are interested in learning about publication distributions in terms of their disciplinary focus: individual-discipline focused versus cross-discipline focused.

**Fig. 2:**
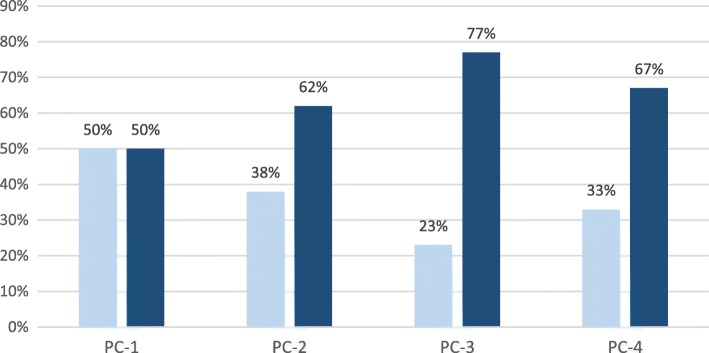
Percentage of publications in single-disciplinary (light color bars) and cross-disciplinary (dark color bars) concentrations for each publication cycle year

Figure 2 shows the percentage distribution of these 118 publications structured by their subject disciplinary concentration: individual- (light color) vs. cross-discipline (dark color), for the four publication cycle years. Overall, 36% of the 118 publications have focused on issues and questions in an individual discipline, and 64% have been cross-disciplinary. Represented among the former are publications on education in a wide variety of individual disciplines, including biology, physics (when individual discipline in science is specified), science (when science is taken as a general discipline), mathematics, and engineering. For those publications in cross-disciplinary concentrations, readers can find publications on issues and questions that span across multiple disciplines, such as science, mathematics, and technology.

The results suggest that the journal has published more articles on cross-disciplinary than single-disciplinary issues and questions, and over the four publication cycle years there has been a generally increasing trend in this regard. These findings confirm that the journal is fulfilling its aim: to provide multidisciplinary perspectives needed to complement individual disciplinary-focused journals in STEM education (Li, 2014). Indeed, the journal has served as a platform for STEM educators and researchers to share their research in individual disciplines at the same place, as opposed to be published across multiple journals. Even more important, STEM educators and researchers have shared their research on issues and questions that are cross-disciplinary in nature. The journal will continue to value and welcome original contributions from different perspectives that view STEM education, either as a collection of traditionally defined, individual-disciplinary-based education separately in S.T.E.M., or as an educational undertaking in inter-connected STEM fields.

## How does the *International Journal of STEM Education* differ from other journals in STEM education, especially the *Journal for STEM Education Research*?

The *International Journal of STEM Education* was established in 2014 as a *multidisciplinary*, open-access, peer-reviewed research journal. It complements individual disciplinary-focused journals in STEM education by “(1) providing an outlet to publish and share research from various disciplines and methods, (2) increasing access to research findings for researchers and educators through the journal’s open-access platform, and (3) galvanizing scientists and educational researchers to further our knowledge about STEM education.” (Li, 2014, p. 1).

The journal’s nature distinguishes it from many other journals in education research, including the newly established *Journal for STEM Education Research (STEM-ER)*, which is also published by Springer (see https://www.springer.com/41979). As noted by Li (2018), these two journals (i.e., the *International Journal of STEM Education* and the *STEM-ER*) complement each other in the following two main regards. First, the *International Journal of STEM Education* is an online open-access journal. Although authors who publish in this journal need to account for article processing charges (APCs), they also enjoy broad free accessibility of their published articles. In contrast, *STEM-ER* is a subscription-based journal that publishes in both print and electronic formats. Although authors who publish in *STEM-ER* do not need to concern about APCs, access to their published articles is restricted to those who pay to subscribe the journal.

The second difference relates to the aim and scope of these two journals. *STEM-ER* is established as an *interdisciplinary* education research journal. It is designed to promote research that helps identifying and addressing sets of basic questions that can not only reflect but also lead the rapid development of integrated STEM education around the globe (Li, 2018). In contrast, the *International Journal of STEM Education* is established as a platform to promote STEM education and research internationally by serving as a *multidisciplinary* education journal that spans disciplinary boundaries. Although interdisciplinary research contributions are preferred, the *International Journal of STEM Education* will remain a gathering place where disciplinary education scholars, who have traditionally been separated, can share both individual-disciplinary-based and cross-disciplinary educational research.

As summarized and discussed above, the *International Journal of STEM Education* has had a great journey over the past four publication cycle years. In many ways, its development mirrors the rapid development of STEM education research around the globe. I expect the journal to continue to grow to further support STEM education research and development. At the same time, I realize that every success of the journal originates from the great contributions and support from numerous authors, scholars, and readers around the world. Thank you all for making the journal an important place to share scholarly information about STEM education and research!

Last but not least, I want to take this opportunity to thank all members of the journal’s editorial board, and staff members at SpringerOpen, for their dedicated support. It is a great pleasure to work together with them.
